# Endophytic fungi associated with *Monarda citriodora,* an aromatic and medicinal plant and their biocontrol potential

**DOI:** 10.1080/13880209.2017.1309054

**Published:** 2017-04-11

**Authors:** Meenu Katoch, Shipra Pull

**Affiliations:** Microbial Biotechnology Department, Indian Institute of Integrative Medicine (CSIR), Jammu, India

**Keywords:** Phytopathogen, fumigation assay, dual culture assay, culture filtrate assay, antagonistic activity

## Abstract

**Context:** The Food and Agriculture Organization has estimated that every year considerable losses of the food crops occur due to plant diseases. Although fungicides are extensively used for management of plant diseases, they are expensive and hazardous to the environment and human health. Alternatively, biological control is the safe way to overcome the effects of plant diseases and to sustain agriculture. Since *Monarda citriodora* Cerv. ex Lag. (Lamiaceae/Labiatae) is known for its antifungal properties, it was chosen for the study.

**Objective:** The isolation of endophytic fungi from *M. citriodora* and assessing their biocontrol potential.

**Material and methods:** The isolated endophytes were characterized using ITS-5.8 S rDNA sequencing. Their biocontrol potential was assessed using different antagonistic assays against major plant pathogens.

**Results:** Twenty-eight endophytes representing 11 genera were isolated, of which, around 82% endophytes showed biocontrol potential against plant pathogens. MC-2 L (*Fusarium oxysporum*), MC-14 F (*F. oxysporum*), MC-22 F (*F. oxysporum*) and MC-25 F (*F. redolens*) displayed significant antagonistic activity against all the tested pathogens. Interestingly, MC-10 L (*Muscodor yucatanensis*) completely inhibited the growth of *Sclerotinia* sp*., Colletotrichum capsici, Aspergillus flavus* and *A. fumigatus* in dual culture assay, whereas MC-8 L (*A. oryzae*) and MC-9 L (*Penicillium commune*) completely inhibited the growth of the *Sclerotinia* sp. in fumigation assay.

**Conclusions:** Endophytes MC-2 L, MC-14 F, MC-22 F and MC-25 F could effectively be used to control broad range of phytopathogens, while MC-10 L, MC-8 L and MC-9 L could be used to control specific pathogens. Secondly, endophytes showing varying degrees of antagonism in different assays represented the chemo-diversity not only as promising biocontrol agents but also as a resource of defensive and bioactive metabolites.

## Introduction

The Food and Agriculture Organization (FAO) has estimated that almost 25% of the world’s crops are affected by plant diseases each year (Schmale & Munkvold 2017). Traditionally, chemical fungicides are used to control the plant diseases caused by fungi, but they are expensive and hazardous for both the environment and human health. Alternatively, biological control is nowadays considered as safe option for plant disease management and to sustain agriculture (Bateman 2002). Biological control mainly relies on the use of antagonistic organisms that broadly/specifically target the pathogen. Antagonistic organisms act on pathogens by various mechanisms, which include mycoparasitism due to physical inter-hyphal interference, competition for nutrients and colonizing space, production of volatiles and nonvolatile metabolites or stimulation of host defence (Dennis & Webster 1971; Larkin et al. 1996; Benhamou et al. 2000; Walters et al. 2005; El-Hasan et al. 2009; Ting et al. 2010). To date, the genus *Trichoderma* Persoon (Hypocreaceae) remains the most studied and widely used economically efficient biological control agent (BCA) against various phytopathogens (Benitez et al. 2004).

Endophytes are the organisms, which colonize in the internal plant tissues without causing any apparent harm to them, and provide resistance against abiotic and/or biotic stresses (Walters et al. 2005). Taxonomically and biologically diverse endophytes have been reported from various wild as well as cultivated crops (Wilson 1995). In past, many endophytes have been evaluated for their biocontrol potential against various plant pathogens (Arnold et al. 2003; Holmes et al. 2004; Ting et al. 2010). Lahlali and Hijri (2010) reported *Trichoderma atroviridae,* an endophyte, as an efficient biocontrol agent for *Rhizoctonia solani* AG3, a causal agent of black scurf on potato.

In the last few decades, the market for microbial/endophytic inoculants with capability to be used as biological control has grown worldwide with an approximately annual growth rate of 10% (Lahlali & Hijri 2010). Thus, it is imperative to investigate biocontrol potential of fungal endophytes, which could be utilized to protect the crops from plant diseases and to increase the crop production.

*Monarda citriodora* Cerv. ex Lag. (Lamiaceae/Labiatae) is commonly called as lemon beebalm or lemon bergamot. It is an erect, widely distributed, medicinal, aromatic and ornamental annual herb (Bailey 1977; Duke 2007). The genus is reported to have origins in the USA, Canada and Mexico and is widely grown in Europe and Asia (Bailey 1977; Collins & Bishop 1993). It consists of roughly 30 species. In India, it has been grown in the Shivalik hills since 1990. There is no report of any fungal disease on *M. citriodora*. Primarily, it is used for variety of purposes such as a flavouring agent and gives delicious flavour to a variety of drinks, bakery and meat products. Due to the presence of citronellol, its leaves are pulverized and sprinkled on meat for its preservation as they repel insects. Its infusion is used to treat human ailments such as catarrh, cold, headache, gastric disorders, tooth ache, fever, flatulence, sore throat, nausea, insomnia and menstrual pain (Bee Balm c2006-2017). It is applied externally to treat skin eruption and infections. Duke (2007) reported its diaphoretic, antirheumatic, carminative, sedative, diuretic, stimulant antibacterial, anticoagulant and antiseptic properties, and its use as a tonic.

*Monarda citriodora* has a rich source of volatile compounds present in its essential oil (Collins et al. 1994; Dorman & Deans 2004; Zhan Guo et al. 2011). Its essential oil is reported to contain 30 constituents. Out of which, four were major ones: β-cymene 4.019%, α-phellandrene 4.815%, 1,8-cineole 23.613% and thymol 44.599% (Zhan Guo et al. 2011). However, Dorman and Deans (2004) reported terpinen-4-ol 1.2%, carvacrol 6.1%, *p*-cymene 10.6%, 7-octen-4-ol and thymol 70.6% as dominant components of its essential oil (95.9%). The essential oil of *M. citriodora* has antibacterial, antioxidant and antifungal properties. It was found to be active against various human pathogens such as *Escherichia coli, Bacillus subtilis, Staphylococcus albus* and many post-harvest pathogens infecting variety of crops (Collins et al. 1993; Bishop & Thornton 1997; Zhan Guo et al. 2011). Recently its essential oil was found to have anticancer property targeting PI3K pathway (Pathania et al. 2013). Thymol, an ingredient of its essential oil has antiseptic properties and is used in modern commercial mouthwash formulations. Thus, the plant has been exploited for its phytochemical and pharmacological activities.

However, the endophytic fungi associated with *M. citriodora* and their role in plant is still unclear and needs to be explored and exploited. The aim of the present study isolates and assesses the biocontrol potential of endophytic fungi associated with different tissues of *M. citriodora* against major fungal phytopathogens using three *in vitro* assays: dual culture, culture filtrate and fumigation assays. The results could be further exploited for the management of plant pathogens and thus reducing the losses caused by plant diseases.

## Materials and methods

### Isolation, identification and phylogenetic characterization of endophytes

Fully mature disease-free healthy plants of *M. citriodora* were collected during March-April, 2013 from the Shivalik hills of Jammu and Kashmir (32.73°N 74.87°E), India. The species was identified by taxonomist (Dr. Bikarma Singh) via leaf and flower morphology and preserved in the *Janaki Ammal Herbarium* (IIIM) (accession no. 18554) and in the farm of IIIM as genetic resource.

The endophytic fungi were isolated from *M. citriodora* (Katoch et al. 2014). Different tissues (leaves, roots and flowers) of the plants were carefully excised with a sterile scalpel. The tissues were thoroughly cleaned by washing in running tap water, followed by deionized (DI) water. Clean tissue pieces were sterilized by keeping them in a series of solution: 70% ethanol; 1.0% sodium hypochlorite (v/v); again 70% ethanol for 1 min in each solution. Finally the tissues were rinsed twice with sterile distilled water to remove extra surfactant. After surface sterilization, tissues were dried on blotting sheets and cut into small pieces of 1 cm^2^. These sterile tissue pieces were placed on Petri plates containing water/potato dextrose agar (WA, PDA) supplemented with streptomycin (250 μg mL^−1^) to inhibit the bacterial growth. The plates were incubated at 25 ± 2 °C and regularly observed for emergence of any fungal growth. The mycelium originating from the tissue in the plates was subcultured on fresh PDA plate. The endophytic fungal isolates so obtained, were coded as per their tissue origin (MC-1 L, MC-2 L, MC-3 L, etc. from leaves, MC-13 R, MC-20 R from roots and MC-7 F, MC-14 F, MC-21 F from flowers). Regular sub culturing was performed after every two months to maintain the cultures. Bits of endophytes were stored in paraffin oil at 4 °C and were deposited in RN Chopra, Microbial Repository, IIIM, Jammu.

ITS-based rDNA sequencing was used to identify the endophytes. Genomic DNA of the endophytes was extracted from the *in vitro* grown biomass of endophytes using the protocol described by Raeder and Broda (1985). Approximately 1 g of dried mycelia was kept in liquid nitrogen and crushed into a fine powder. It was transferred to 10 mL of extraction buffer and vortexed thoroughly. The samples were incubated in water bath set at 65 °C for 30 min with intermittent mixing. The tubes were centrifuged at 10,000 *g* for 5–10 min followed by extraction of aqueous layer with chloroform:isoamyl alcohol (24:1). Aqueous layer was collected and DNA was precipitated with 2.5–3 volume of absolute ethanol in the presence of 1/10th volume of sodium acetate. Tubes were inverted slowly to mix the contents and centrifuged at 8000 *g* for 20 min at 4 °C. So obtained white/transparent pellets were washed with ice-cold 70% ethanol followed by air drying. Dried pellets were dissolved in 20 μL of water (molecular biology grade). ITS sequences containing ITS1-5.8 S-ITS2 region spanning 500–600 bp were amplified with the universal primers ITS1 and ITS4 (White et al. 1990). PCR reaction was set up in 50 μL containing DNA (1–10 ng), 1× PCR buffer (with 15 mM MgCl_2_), each dNTP (200 mM), each primer (10 pmol, Sigma) and 1 U *Taq* DNA polymerase (Bangalore Genei, India). Cycling parameters were 5 min at 94 °C followed by 30 cycles at 94 °C for 30 s, 55 °C for 1 min, 72 °C for 1 min and a final extension for 10 min at 72 °C. The PCR product (10 μL) was resolved using agarose gel electrophoresis at 100 V. The amplified product was purified using a Gel Extraction Kit (Qiagen) and sequencing reaction was set up in a 10 μL: 40–60 ng of purified PCR product, 3.2 pmol forward/reverse primer, Big Dye Terminator sequencing mix 8 μL (v. 3.1, Applied Biosystems, US). Samples were sequenced on an automated sequencing system (Applied Biosystems). Resultant sequences (KU527781-KU527806, KU680345) were submitted to the gene bank. These sequences were blasted against the nucleotide database using BLASTn Tool of the National Centre for Biotechnology Information (NCBI), US to identify the endophytes (Altschul et al. 1997).

### Biocontrol potential of endophytes

#### Fungal pathogens used

Antagonistic activity of all the fungal endophytes isolated from *M. citriodora* was evaluated against common phytopathogens namely, *Aspergillus flavus* (accession number MTCC 1783), *F. solani* (MTCC 350), *Sclerotinia* sp. (MTCC 7114)*, Colletotrichum capsici* (MTCC 2071)*, A. fumigatus* (MTCC 343), procured from The Microbial Type *Culture* Collection and Gene Bank (*MTCC*), India. The fungal cultures were revived and regularly subcultured on potato dextrose agar (PDA) under aseptic conditions as per the guidelines given by *MTCC*, India.

#### Antagonistic studies

Antagonistic potential of the endophytic fungal isolates was assessed through dual culture assay, culture filtrate assay and fumigation assay (Lahlali et al. 2007; Miles et al. 2012). All the experiments were performed in triplicate and mean values were taken.

##### Dual culture assay

Dual culture assay was used for comparative evaluation of the endophytes for their ability to inhibit the fungal pathogen’s growth on potato dextrose agar (PDA) (Lahlali et al. 2007). Petri plates containing 15–20 mL potato dextrose agar (PDA) were prepared. Bits of isolated endophyte and pathogen (0.5 cm each) were co-cultured at the two opposite ends of the plates. The plates were incubated at 25 ± 2 °C after sealing them with parafilm. The pathogens alone (at one end of plate) without endophyte were served as control. After 7 days, the radial growth of pathogen in the presence/absence of the fungal endophyte was measured. The percent antagonism was calculated using the formula A (%) = [(CDC-CDT)/CDC] × 100, where CDC represents the colony radial growth of pathogen (measured in mm) on the control plates i.e., in the absence of endophyte and CDT is the radial growth of pathogen in test plate i.e., in the presence of endophyte (Ezra et al. 2004; Chamberlain & Crawford 1999).

##### Culture filtrate assay

Each endophyte was cultured in 50 mL of potato dextrose broth (PDB) in 250 mL Erlenmeyer flasks by inoculating two plugs (0.5 cm) of actively growing endophytic fungus. The flasks were incubated for 10 days in rotatory shaker (150 rpm) at 25 ± 2 °C. The cultures were centrifuged to remove biomass so as to collect the broth containing antagonist metabolites (Dennis & Webster 1971). Endophytic culture filtrate (200 μL) was spread on PDA plates to test their antagonistic activity. On drying of filtrate, respective pathogenic fungus (0.5 cm plug) was inoculated at the centre of PDA plate. Simultaneously PDA plates containing pathogenic fungi without endophytic culture filtrate served as control. The plates were incubated at 25 ± 2 °C. After 7 days, the radial growth of pathogen in the presence/absence of endophytic culture filtrate was monitored and percent antagonism was calculated for respective culture filtrate.

##### Fumigation assay

The assay was conducted in PDA Petri plates, which were inoculated separately with endophytic and pathogenic cultures (0.5 cm plug from actively growing fungus). The plates were incubated at 25 ± 2 °C after sealing with parafilm. After 2–4 days, lids were removed and Petri dish inoculated with endophytic culture was inverted on the petri dish inoculated with pathogen under aseptic conditions and were tightened with double layer of parafilm. This creates no physical contact between the agar containing pathogen and endophyte. Plates were incubated at 25 ± 2 °C for 7 days. Petri dish containing PDA without any endophyte inverted on the Petri dish inoculated with pathogen served as control. Radial growth of pathogen in the presence/absence of endophyte culture was monitored and percent antagonism was calculated for each antagonist-pathogen combination.

### Clustering of endophytes with biocontrol potential

For clustering the endophytes with biocontrol potential, a tree was generated using NTSYS program. Results of biocontrol potential were converted into binary form of data. Endophytes inhibiting more than 50% growth of pathogen were designated as 1, whereas those inhibiting less than 50% growth of pathogen were given as 0. Data were compiled for all the isolated endophytes against all the tested plant pathogens in three different assays. These data sets were used to generate the tree.

## Results

### Identification and characterization of the endophytic fungi

Twenty-eight endophytic fungi were isolated from healthy and symptomless tissues (leaves, roots and flowers) of *M. citriodora* to determine their biocontrol potential. Their morphology and growth characteristics on potato dextrose agar were recorded (Supplementary file Figure S1; Table S1). Their molecular identification was carried out on the basis of rDNA sequences. The ITS sequences of each endophyte were blasted against the nucleotide database of NCBI for the most homologous sequence. The endophytes were assigned as particular species only if minimum threshold similarity was ≥99% compared to the most closely related strain (Yuan et al. 2010). In the present study, only two sequences (for isolates MC-8 L and MC-20 L) showed <99% similarity to the known sequences. Details of the fungal endophytes, GenBank accession numbers and closest sequence homologs are given in Table 1.

**Table 1.** Fungal endophytes isolated from various tissues of *M. citriodora* with their respective strain codes, Gen-Bank accession numbers and closest affiliations of the representative isolates in the GenBank according to rDNA ITS analysis.

S No.	Endophyte	EMBL-Bankaccession number	Most closely related strain(accession number)	Maximumidentity (%)
1	MC-1L	KU527781	*C. boninense* JQ676184	100
2	MC-2L	KU527782	*F. chlamydosporum* KP641161	99
3	MC-3L	KU527783	*C. aeria* KP131939	99
4	MC-4L	KU527784	*A. alternata* GQ121322	100
5	MC-5L	KU527785	*A. flavus* KM285408	99
6	MC-6L	KU527786	*N. hiratsukae* GQ461906	98
7	MC-8L^a^	KU680345	*A. oryzae* KT964480	93
8	MC-9L	KU527787	*P. commune* KF938402	99
9	MC-10L	KU527788	*M. yucatanensis* KJ572191	99
10	MC-12L	KU527789	*F. solani* FJ426390	99
11	MC-14L	KU527790	*F. oxysporum* JX406507	99
12	MC-15L	KU527791	*Aspergillus* sp. GQ352493	99
13	MC-16L	KU527792	*Neurospora* sp. KJ676544	99
14	MC-17L	KU527793	*A. waksmanii* EF669934	99
15	MC-18L	KU527794	*A. fumigatus* KM207771	99
16	MC-20L^a^	KU680346	*Cladosporium* sp. KP050606	89
17	MC-24L	KU527795	*C. tenuissimum* KJ589554	100
18	MC-25L	KU527796	*Fusarium* sp. KC007281	100
19	MC-13R	KU527797	*A. carthami* JF710542	99
20	MC-20R	KU527798	*Cladosporium* sp. JQ388271	99
21	MC-7F	KU527799	*Fusarium* sp. KJ567458	99
22	MC-14F	KU527800	*F. oxysporum* KT876658	99
23	MC-17F	KU527801	*C. gloeosporioides* KM520010	99
24	MC-21F	KU527802	*C. cladosporioides* KP900248	99
25	MC-22F	KU527803	*F. oxysporum* KF264963	100
26	MC-23F	KU527804	*G. intermedia* JQ846048	99
27	MC-25F	KU527805	*F. redolens* KJ540090	99
28	MC-26F	KU527806	*F. oxysporum* KF998987	99

a Homology is less but it was supported by morphology.

The results of blast analysis revealed that the endophytic fungi belonged to the phylum Ascomycota. Out of these, 46.4% of endophytic fungi belonged to the class Sordariomycetes followed by Eurotiomycetes (28.5%), and Dothideomycetes (25%). Thus, Sordariomycetes was the most abundant class of fungi, which was represented by the orders Hypocreales, Xylariales, Glomerellales and Sordariales. They are mostly distributed in leaves and flowers. Eurotiomycetes were isolated only from leaves and represented by the order Eurotiales, whereas Dothideomycetes were isolated from all the tissues and are represented by the order Pleosporales and Capnodiales. The isolated endophytic fungi belonged to 11 different genera. The dominant fungi observed were *Fusarium* spp., *Aspergillus* spp. *Cladosporium* spp. In leaves, *Aspergillus* spp. (27%) showed the highest isolation frequency followed by *Fusarium* spp. (22%) and *Cladosporium* spp. (11%), while in flowers, *Fusarium* spp. (62.5%) showed the highest isolation frequency. *Fusarium* spp. were common in leaves and flowers, whereas *Cladosporium* sp. was common in leaves and roots. Endophytes isolated only from the leaves tissues were *Curvularia aeria, Neosartorya hiratsukae, M. yucatanensis, Neurospora* sp*., C. boninense, Alternaria alternata, Penicillium commune, Fusarium* spp*., Aspergillus* spp. and *Cladosporium* spp., whereas *Gibberella intermedia, Fusarium* spp*., C. cladosporioides*, and *C. gloeosporioides* were restricted to flowers and roots were observed to harbour only *A. carthami* and *Cladosporium* spp.

### *In vitro* antagonistic activity of endophytes

All the endophytic strains were assayed for antagonistic activity against major phytopathogens using three different assays. The percentage antagonism (growth inhibition percentage) was calculated for each endophyte and summarized in Table 2 (dual culture assay), Table 3 (culture filtrate assay) and Table 4 (fumigation assay). Percentage antagonism varied significantly among endophytes investigated through different assays, which supported the OSMAC hypothesis (Bode et al. 2002). These endophyte–pathogen interaction studies gave an insight to understand the correlation between diversity of micro-flora associated with the plant tissues and their chemo-diversity.

**Table 2.** Biocontrol potential of endophytes against plant pathogens using dual culture assay in terms of percent growth inhibition.

S No.	Endophyte	*F. solani*	*Sclerotinia* sp.	*Colletotricum capsici*	*A. flavus*	*A. fumigatus*
1	MC-1-L	–	47.6	–	–	–
2	MC-2-L	**59.6**	**69.9**	**78.4**	**73.4**	**74**
3	MC-3-L	–	22.8	**53.2**	–	–
4	MC-4-L	–	43	–	–	**66.2**
5	MC-5-L	39.1	–	**73.2**	–	–
6	MC-6-L	–	–	–	–	–
7	MC-8-L	–	–	–	–	–
8	MC-9-L	–	–	–	–	–
9	MC-10-L	–	**100**	**100**	**98**	**100**
10	MC-12-L	–	–	**78.2**	–	–
11	MC-14-L	9.5	17.3	–	40.2	28.2
12	MC-15-L	11.5	11.3	–	17.07	4.8
13	MC-16-L	15.07	41	**78.1**	29.7	41.5
14	MC-17-L	–	–	**-**	–	–
15	MC-18-L	–	–	**78.7**	–	–
16	MC-20-L	–	42	20	–	35
17	MC-24-L	26.9	**50**	–	3.5	36.8
18	MC-25-L	31.2	–	–	28.6	29.4
19	MC-13-R	–	**78.9**	**51**	**62.7**	43.1
20	MC-20-R	41.6	39.8	–	–	–
21	MC-7-F	5.2	–	–	0.61	44.15
22	MC-14-F	**56.2**	34	**52.6**	–	–
23	MC-17-F	–	40	15.09	**62.4**	–
24	MC-21-F	24.6	40	22	46.8	0.6
25	MC-22-F	–	–	**59.4**	–	–
26	MC-23-F	–	3.4	–	–	38.4
27	MC-25-F	**52.3**	**64.1**	**72.3**	–	–
28	MC-26-F	6.3	24.05	**54.12**	10.08	42.9

>50% inhibition is in bold.

**Table 3.** Biocontrol potential of endophytes against plant pathogens using culture filtrate assay in terms of percent growth inhibition.

S No.	Endophyte	*F. solani*	*Sclerotinia* sp.	*C. capsici*	*A. flavus*	*A. fumigatus*
1	MC-1-L	25	15	14.28	10	35
2	MC-2-L	4	20	50	50	50
3	MC-3-L	25	20	21.42	15	30
4	MC-4-L	**50**	**69.56**	**63.15**	**80**	44
5	MC-5-L	33.33	**65.21**	**68.42**	**65**	44
6	MC-6-L	**50**	**56.52**	**73.68**	**60**	22.22
7	MC-8-L	**50**	**60.87**	21.05	**50**	22.22
8	MC-9-L	**56.66**	**56.52**	47.36	30	**55**
9	MC-10-L	25	20	14.28	15	40
10	MC-12-L	29.16	10	**50**	20	10
11	MC-14-L	**66**	**75**	28.57	**75**	25
12	MC-15-L	**56.66**	34.78	21.05	35	**50**
13	MC-16-L	16	25	21.42	**55**	20
14	MC-17-L	33.33	47.82	**57.89**	10	44
15	MC-18-L	**56.66**	**65.21**	5.2	**75**	5
16	MC-20-L	25	10	7.1	5	5
17	MC-24-L	**53.33**	43.47	**52.63**	**55**	22.22
18	MC-25-L	46.66	**65.21**	47.36	**50**	11
19	MC-13-R	16	40	21.42	**90**	20
20	MC-20-R	16	0	28.57	25	35
21	MC-7-F	29.16	**50**	42.85	**50**	40
22	MC-14-F	**66.66**	**69.56**	**63.15**	**65**	**72.2**
23	MC-17-F	25	15	42.85	**60**	10
24	MC-21-F	25	20	0	10	**50**
25	MC-22-F	**56.66**	**65.21**	**57.89**	**65**	**61.1**
26	MC-23-F	25	40	7.1	20	20
27	MC-25-F	**56.66**	**60.87**	**57.89**	**60**	**55**
28	MC-26-F	**50**	**73.91**	10.52	**65**	27

>50% inhibition is in bold.

**Table 4.** Biocontrol potential of endophytes against plant pathogens using fumigation assay in terms of percent growth inhibition.

S No.	Endophyte	*F. solani*	*Sclerotinia* sp.	*C. capsici*	*A. flavus*	*A. fumigatus*
1	MC-1-L	2	47.91	33.33	**71.05**	**71.11**
2	MC-2-L	0	16.67	27.77	26.82	37.77
3	MC-3-L	33.33	39.58	27.77	22.22	39.74
4	MC-4-L	16	20.83	33.33	4.87	**68.88**
5	MC-5-L	23.33	21.05	0	0	48.88
6	MC-6-L	6	0	16.66	46.34	**55.55**
7	MC-8-L	10	**100**	**60**	**75.61**	**66.66**
8	MC-9-L	33.33	**100**	**72.22**	**51.22**	**55.55**
9	MC-10-L	40	0	**83.33**	**88**	**68.88**
10	MC-12-L	16	**68.75**		34.14	**51.11**
11	MC-14-L	27.08	**73.68**	22.22	36.58	40
12	MC-15-L	40	41.67	33.3	**56.09**	33.33
13	MC-16-L	6	43.75	**72.22**	26.82	46.66
14	MC-17-L	0	**52.08**	27.77	0	33.33
15	MC-18-L	20.8	26.31	**72.22**	39.02	**51.11**
16	MC-20-L	23.33	31.15	44.44	7.31	0
17	MC-24-L	47.91	36.84	44.44	37.5	40
18	MC-25-L	36.6	**50**	**0**	**52**	24.44
19	MC-13-R	10.41	8.33	27.77	43.9	11.11
20	MC-20-R	0	35.41	44.44	21.95	**55.55**
21	MC-7-F	10	10.52	33.33	26.82	48.88
22	MC-14-F	10	36.84	38.88	7.3	40
23	MC-17-F	10	0	55.55	39.02	26.66
24	MC-21-F	3	**58.33**	**61.11**	39.02	0
25	MC-22-F	25	0	27.77	**60**	28.88
26	MC-23-F	0	35.41	44.44	25	33.33
27	MC-25-F	6	47.91	**88.88**	26.82	40
28	MC-26-F	75	33.33	16.66	39.02	22.22

>50% inhibition is in bold.

Growth inhibition ≥50% against one or more pathogens was recorded by 61% of the endophytes using dual culture assay followed by 71% and 82% endophytes using fumigation assay and culture filtrate assay, respectively (Figure S2). In dual culture and fumigation assay, percentage of growth inhibition by different endophytes was ranged between 50–100%, whereas in culture filtrate assay, it was ranged between 50–90%. The eight endophytes (MC-4 L, MC-12 L, MC-16 L, MC-18 L, MC-17 F, MC-22 F, MC-25 F and MC-26 F) inhibited the growth (≥50%) of one or more plant pathogens in all the three assays. The endophytes, which showed growth inhibition (≥50%) in particular assay against one or more plant pathogens were MC-3 L (Dual culture assay), MC-7 F (culture filtrate assay) and MC-1 L and MC-20 R (fumigation assay). Endophytes MC-2 L, MC-5 L, MC-24 L, MC-13 R and MC-14 F inhibited the growth (≥50%) of one or more plant pathogens in both dual culture and culture filtrate assay, whereas endophytes MC-6 L, MC-8 L, MC-9 L, MC-14 L, MC-15 L, MC-17 L, MC-25 L and MC-21 F inhibited the growth (≥50%) of one or more plant pathogens in both culture filtrate and fumigation assay. Endophyte MC-10 L inhibited the growth (≥50%) of one or more plant pathogens in both dual culture and fumigation assay.

Endophytes MC-2 L (*F. chlamydosporum*), MC-14 F (*F. oxysporum*), MC-22 F (*F. oxysporum*) and MC-25 F (*F. redolens*) displayed broad range of antagonistic activity i.e., active against all the plant pathogens tested. Interestingly, the endophytic fungal strain MC-10 L completely inhibit the growth of the *Sclerotinia* sp*., C. capsici, A. flavus,* and *A. fumigatus* in dual culture assay (Figure S2), whereas the endophytic fungal strain MC-8 L, and MC-9 L completely inhibited the growth of the *Sclerotinia* sp*.,* in fumigation assay. In culture filtrate assay, no endophyte could completely inhibit the growth of phytopathogens.

In dual culture assay, the most susceptible pathogen was found to be *C. capsici,* whose growth (≥50%) was inhibited by most of the endophytic fungi MC-2 L, MC-3 L, MC-5 L, MC-10 L, MC-12 L, MC-16 L, MC-18 L, MC-13 R, MC-14 F, MC-22 F, MC-25 F and MC-26 F, whereas in culture filtrate and fumigation assays, the most susceptible pathogen was found to be *A. flavus* and *A. fumigatus*, respectively. The most resistant pathogen was found to be *A. fumigatus, A. fumigatus* and *Fusarium* sp. in dual culture, culture filtrate and fumigation assay, respectively.

### Clustering analysis

**Figure 1.**
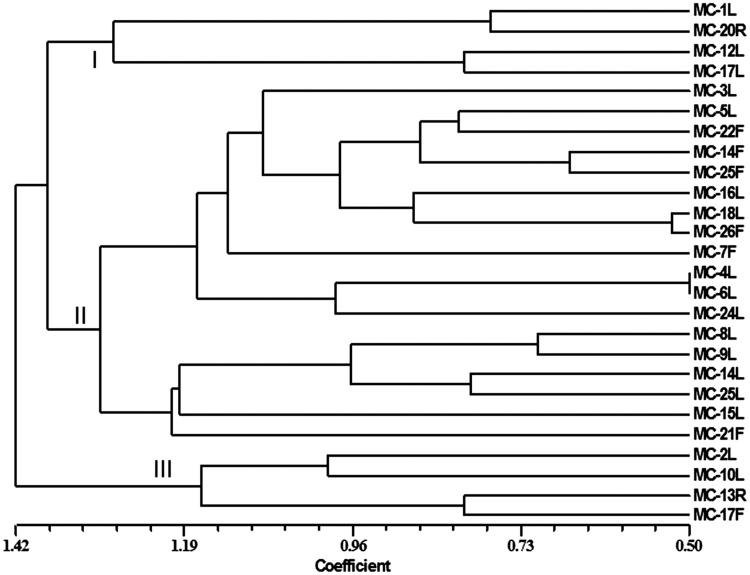
Phylogenetic tree generated using NTSYS program showing the clustering of endophytes with varying degree of antagonism.

For clustering the endophytes with biocontrol potential, a tree was generated, which resulted into three major groups (Figure 1). First and third group contained four endophytes each while the second group contained rest of the endophytes.

## Discussion

Endophytes have been characterized and preserved from diverse plants but still huge part of this natural treasure remains unexplored. Ecosystem plays a vital role in the process of endophytism or vice versa through host disease resistance, adaptation to unique niches, plant secondary metabolism etc. (Porras & Bayman 2011; Singh et al. 2011). Although, there is no genuine model available to study its various aspects including host specificity, geographical distribution and phylogenetic affinities, still different strategies have been adopted to study the endophytes. One of the model emphasized detailed investigation of endophytes from randomly collected plants followed by the study of ecosystem. Another model includes establishment of evolutionary relationships between plants at a particular site through morphological and molecular data, followed by characterization of endophytes from the desired plants. Pharmacologically useful plants could also be the legitimate target to explore the endophytes (Debbab et al. 2012). Many of the aromatic plants have medicinal potential but they are under studied in terms of the presence of endophytes. This present study is therefore the first report of endophytes associated with *M. citriodora* and their biocontrol potential.

There is no report of fungal disease infecting *M. citriodora.* This suggests that either plant’s defence system is quite strong or its endophytes manage to control the infection of plant pathogen. To understand the involvement of endophytes in biocontrol, their antagonistic behaviour was studied against major phytopathogens using different assay techniques. Consecutively in two assays, endophytes were grown on solid and liquid potato dextrose medium, whereas in third assay only volatiles metabolites of endophytes were evaluated against plant pathogens. In our experiments, varying growth inhibitory percentages were observed suggesting the chemo-diversity of endophytic fungi under three different assay conditions. These results were found in agreement with the concept of OSMAC in different culturing conditions; different molecules are produced by microbes (Bode et al. 2002). Kusari et al. (2013) also observed varying degree of antagonistic behaviour on five different growth media (Sabouraud agar, malt extract, Potato dextrose, water agar, nutrient agar) against two host plant pathogens. Our results are in concurrence with Paranagama et al. (2007), who reported that *Chaetomium chiversii* (J.C. Cooke) A. Carter (Chaetomiceae) produces the radicicol instead of chaetochromin A while changing the solid medium to liquid medium. The present study revealed that change in media, growth conditions and/or challenging it with plant pathogen might have elicited the endophytes for the production of ‘cryptic’ metabolites.

In the present study, endophytes MC-2 L (*F. chlamydosporum*), MC-14 F (*F. oxysporum*), MC-22 F (*F. oxysporum*) and MC-25 F (*F. redolens*) displayed broad range of antagonistic activity. Interestingly, the endophytic fungal strain MC-10 L completely inhibit the growth of the *Sclerotinia* sp*., C. capsici, A. flavus* and *A. fumigatus* in dual culture assay, whereas the endophytic fungal strain MC-8 L, and MC-9 L completely inhibited the growth of the *Sclerotinia* sp. Six endophytes 4 L (*A. alternata*), 8 L (*A. oryzae*), 9 L (*P. commune)*, 10 L (*M. yucatanensis*), 13 R (*A. carthami*) and 25 F (*F. redolens*) of the present study showing more than 80% growth inhibition against the plant pathogenic fungi were also considered promising (Table 1).

For clustering the endophytes with biocontrol potential, a tree was generated, which resulted into three major groups. Four endophytes with lowest biocontrol potential were categorized in the first group, while another four endophytes with broad spectrum biocontrol potential were placed in the third group. The second group contained rest of the endophytes with medium range of biocontrol potential against one/two plant pathogens. It is a well-known fact that endophytes produce variety of natural products, which are basically responsible for the biocontrol potential. These grouping also indicate about the molecules active against plant pathogens. It can be speculated that group III produced highest number of bioactive molecules or molecules active against many plant pathogens, while group I produced minimum bioactive molecules or molecules with no biocontrol potential.

Endophytes typically protects the plants by various mechanisms such as production of antifungal compounds, outcompete or displace pathogens, and induces cell death in the pathogen by disrupting pathogen’s cellular membranes (Ganley et al. 2008; Shittu et al. 2009). Endophytic fungi have been source of various bioactive secondary metabolites (Gao et al. 2010). These metabolites might have involved in controlling the pathogenic incidences. Ownley et al. (2010) also observed that *Beauveria* spp. produces a battery of bioactive metabolites and restrict the growth of fungal plant pathogens under *in vitro* conditions, which was in agreement with our results. In this study, *Fusarium* spp., *A. oryzae*, *P. commune, Alternaria* spp. and *M. yucatanensis* endophytes showed promising biocontrol potential ability. These fungi produce variety of natural products (Firakova et al. 2007; Brakhage 2013). These natural products either triggers the cell death in the pathogen or lowers the metabolism of pathogens required for its further growth. Use of these bioactive ingredients as biocontrol agent would be cumbersome and costlier because it requires purification of active ingredient from the complex mixture of bio-molecules present in fermented culture of endophyte. Earlier researches also reported the inoculation of fungal endophytes to plants for mitigation of pathogenic diseases (Arnold et al. 2003; Mejia et al. 2008; Lee et al. 2009).

Thus, these endophytes could directly be used to manage plant diseases under field conditions. To understand the biocontrol potential scientifically and pin pointing the active principle, we have started suitable devised fermentation of endophytes with challenging pathogens. Concerted efforts in this direction would in turn elucidate the mechanism behind it.

## Supplementary Material

Meenu_Katoch_et_al_supplemental_content.zip
