# Supplementary material for: Endophytic fungi associated with Monarda citriodora, an aromatic and medicinal plant and their biocontrol potential
Source: Pharm Biol. 2017 Apr 11;55(1):1528–35. doi: 10.1080/13880209.2017.1309054 (PMC6130496; doi:10.1080/13880209.2017.1309054)
Supplement: Meenu_Katoch_et_al_supplemental_content.zip [file IPHB_A_1309054_SM1041.zip › Meenu Katoch et al supplemental content.doc]

**Endophytic fungi associated with *Monarda citriodora* L., an aromatic and medicinal plant and their biocontrol potential**

Meenu Katoch^1*^, Shipra Pull^1^

^1^Microbial Biotechnology Department

Indian Institute of Integrative Medicine (CSIR), Jammu, 180001, India

**Fig S1** Growth of endophytes isolated from leaves, roots and flowers of *Monarda citriodora* L. on potato dextrose agar plates

MC-1-L

MC-2-L

MC-3-L

MC-4-L

MC-5-L

MC-6-L

MC-7-F

MC-8-L

MC-9-L

MC-10-L

MC-11-L

MC-12-L

MC-13-R

MC-14-L

MC-14-F

MC-17-F

MC-16-L

MC-15-L

MC-18-L

MC-20-L

MC-20-R

MC-21-F

MC-22-F

MC-23-F

MC-26-F

MC-25-L

MC-24-L

**Table S1: Morphological Characteristics of endophytes isolated from *Monarda citriodora* L. grown on potato dextrose agar at 25 ± 2°C for 10 days with no light**

| **Endophyte** | **Colour of mycelium** | **Morphology** |
| --- | --- | --- |
| MC-1-L | orangish | Orange filaments with black spores |
| MC-2-L | brownish | highly filamentous |
| MC-3-L | dark black | filamentous |
| MC-4-L | Dark green | Highly sporogenous |
| MC-5-L | black | filamentous |
| MC-6-L | green and white | alternate patches of green and white |
| MC-7-F | white and pink | highly filamentous |
| MC-8-L | Parrot green | highly sporogenous |
| MC-9-L | green (in patches) | highly sporogenous |
| MC-10-L | White | filamentous |
| MC-11-L | Blackish | highly sporogenous |
| MC-12-L | Orangish brown | velvety |
| MC-13-R | Grey with orange background | Velvety and filamentous |
| MC-14-L | Oranges with brown background | slightly filamentous |
| MC-14-F | pink | filamentous |
| MC-15-L | Orangish brown | filamentous |
| MC-16-L | white | Sticky and velvety |
| MC-17-L | sea green | velvety |
| MC-17-F | Black | Sporogenous |
| MC-18-L | green | Highly sporogenous |
| MC-20-L | Dark green | Filamentous |
| MC-20-R | Highly blackish green | Sporogenous |
| MC-21-F | Dark green | Sporogenous |
| MC-22-F | Pink and purple | Filamentous |
| MC-23-F | Pink and purple concentric rings | Filamentous |
| MC-24-L | Green and orange | Highly sporogenous |
| MC-25-F | Purple and white | Filamentous |
| MC-25-L | Pink and white | Filamentous |
| MC-26-F | pink | Filamentous |

**Fig S2**: Endophyte pathogen interaction observed in dual culture antagonistic assay. Plate on left are showing challenging pathogen without endophytes, while plates showing on right have both endophyte and challenging pathogens on same plate but at middle of their half sector a) MC-10L *Colletotricum capsici* b) MC-10L *Sclerotinia* sp*.*
